# Pharmacological Characteristics of the Hydroethanolic Extract of *Acmella oleracea* (L) R. K. Jansen Flowers: ADME/Tox *In Silico* and *In Vivo* Antihypertensive and Chronic Toxicity Evaluation

**DOI:** 10.1155/2023/1278720

**Published:** 2023-04-29

**Authors:** Emanuelle T. Rodrigues, Paulo Peretti, Roberto M. Bezerra, Manoel F. Biancardi, Francisco F. O. Sousa, Elizabeth P. Mendes, João B. R. Dutra, Carla C. R. Silveira, Carlos H. Castro, Jorddy N. Cruz, Cleydson B. R. Santos, Fernanda C. A. Santos, Mayara T. Pinheiro

**Affiliations:** ^1^Laboratory of Biotechnology in Natural Products, Faculty of Pharmacy, Department of Biological and Health Sciences, Federal University of Amapá, Macapá, Amapá, Brazil; ^2^Graduate Program in Health Sciences, Department of Biological and Health Sciences, Federal University of Amapá, Macapá, Amapá, Brazil; ^3^Laboratory of Atomic Absorption and Bioprospecting, Department of Biological and Health Sciences, Federal University of Amapá, Macapá, Amapá, Brazil; ^4^Department of Histology, Embryology and Cell Biology, Laboratory of Microscopy Applied to Reproduction, Institute of Biological Sciences, Federal University of Goiás, Goiânia, Goiás, Brazil; ^5^Laboratory of Quality Control and Bromatology, Faculty of Pharmacy, Department of Biological and Health Sciences, Federal University of Amapá, Macapá, Amapá, Brazil; ^6^Department of Physiological Sciences, Institute of Biological Sciences, Federal University of Goiás, Goiânia, Goiás, Brazil; ^7^Integrated Laboratory of Cardiovascular and Neurological Pathophysiology, Federal University of Goiás, Goiânia, Goiás, Brazil; ^8^Laboratory of Modeling and Computational Chemistry, Department of Biological and Health Sciences, Federal University of Amapá, Macapá, Amapá, Brazil

## Abstract

*Acmella oleracea* (L.) R. K. Jansen, popularly known as jambu in Northern Brazil, is widely used in folk medicine and local cuisine. Its consumption in different ways reinforces the need for safety assessments. In this study, the major compounds found in the hydroethanolic extract of *A. oleracea* flowers (EHFAO) were characterized by ultra-performance liquid mass spectrometry (UHPLC-ESI-QTOF-MS/MS). The effects of oral administration of 100/mg/kg of EHFAO extract over 60 days in male spontaneously hypertensive (SHR) and Wistar (WR) rats and the *in silico* ADME/Tox predictions, lipophilicity, and water solubility were accomplished for the compounds identified. Spilanthol was detected as the foremost major compound at a concentration of 97.7%, followed by 1.53% scopoletin and 0.77% d-limonene. The treatment with EHFAO did not alter the animals´ weight over the studied period. Moderate alterations were observed solely in the hepatic enzymes AST (WR = 97 UI/L and SHR = 150 UI/L ^*∗*^*p* < 0.05) and ALT (WR = 55 UI/L and SHR = 95 UI/L ^*∗*^*p* < 0.05), while no relevant histopathological alterations were found. The *in-silico* study confirmed the *in vivo* findings, as the identified compounds were considered highly bioactive orally, due to their drug similarity profiles, adequate lipid solubility, bioavailability, and pharmacokinetics. Therefore, the chronic treatment with EHFAO was found safe at the concentration of 100/mg/kg, with no interference in the blood pressure levels neither appreciable toxic effects.

## 1. Introduction

**Figure 1:**
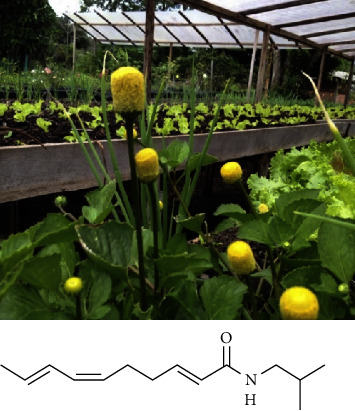
*Acmella oleracea* detail of flowers and leaves and chemical structure of spilanthol (N-isobutyl-2(E),6(Z),8(E)-decatrienamide.

*Acmella oleracea* (L.) R. K. Jansen, popularly known as jambu (Figure 1), is an edible plant widely used in the Amazonian cuisine and folk medicine. Belonging to the Asteraceae family, it is native in Eastern Amazon and cultivated in the Brazilian states of Pará and Amapá [1, 2]. It has demonstrated important pharmacological properties such as anti-inflammatory, diuretic, vasorelaxant, antioxidant, and antibacterial [3, 4].

The most representative chemical compounds found in this species are alkylamides, especially spilanthol ((2E,6Z,8E)-N-Isobutyl 2,6,8-decatrienamide) (Figure 1), also known as Aphnin [5], abundantly found in the leaves, stems, and flowers [6, 7]. Several biological activities are attributed to spilanthol, such as analgesic, antibacterial [8], antinociceptive [9], anti-inflammatory [10], and anesthetic [11]. The higher spilanthol content found in *A. oleracea* flowers, together with the other constituents, can mask the taste, making it commonly unusable for cooking [12, 13].

Pharmacotoxicological research with medicinal plants, either *in vitro*, preclinical, and/or clinical, is aimed at improving the understanding over their effects, ensuring that derived products are effective and safe [14, 15]. Nonetheless, few studies regarding the toxicity of *A. oleracea* consumption are reported in the literature. Rocha et al.[14] studied the effect of the hydroethanolic extract of *A. oleracea* flowers on the reproductive toxicity of WR; the authors concluded that the safe use of the extract altered the estrous cycle, without alterations in the folliculogenesis and fertility, though. Therefore, it is crucial to carry out toxicological studies using different models and targets that demonstrate the safety of *A. oleracea* consumption. Molecular modeling approaches have also been successfully used to achieve this goal [16–18].

Accordingly, this study aimed to characterize the composition of EHFAO and evaluate the pharmacokinetics and toxicology *in silico* and its antihypertensive effect and chronic toxicity *in vivo*.

## 2. Results and Discussion

### 2.1. Phytochemical Characterization of the Hydroethanolic Extract of *A. oleracea* Flowers

Spilanthol (2E,6Z,8E)-N-isobutyl-2,6,8-decatrienamide) (Figure 1) is foremost the major phytochemical found in *A. oleracea* (Figure 1(a)) [8, 19]. The UHPLC-ESI-QTOF-MS/MS analysis of the extract showed an evident peak at 6.9 minutes, corresponding to spilanthol in the total ion chromatogram (TIC). Peretti et al. [19] used the same technique to evaluate the extracts of two subtypes of *A. oleracea* and found the same retention time for this compound.

**Figure 2:**
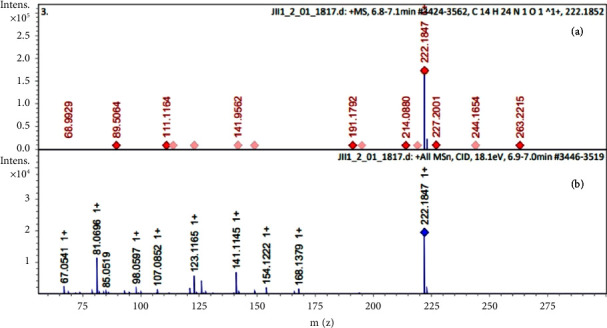
Spilanthol spectrum (a) from the hydroethanolic extract of *Acmella oleracea* flowers showing an *m*/*z* ratio of 222.1847. Very close value compared to the standard spilanthol spectrum and (b) with an *m*/*z* of 222.1852.

The coupling to the mass spectrometer allows obtaining traces of the charge/mass ratio, increasing the accuracy in identifying molecules. The values found in this study are within this range, although with greater sensitivity due to the use of the QToF-MS/MS detector, which makes it possible to differentiate similar molecules with greater precision. The spilanthol identified in the EHFAO had an *m*/*z* of 222.1847 (Figure 2(a)), very similar to that found on its simulated standard spectrum (222.1847) (Figure 2(b)), corroborating also the data obtained by Peretti et al. [19].

The data obtained after analyzing the EHFAO by UHPLC-MS-MS are displayed in Table 1. The extractive yield was 1.82%, while spilanthol represented 97.7% of the extractive content, followed by scopoletin (1.53%) and d-limonene (0.77%).

### 2.2. Oral Bioavailability *In Silico* (Rule of Five: Lipinski)

The compounds identified were submitted to the *in silico* model of bioavailability to evaluate the size of the molecules. The compounds found in EHFAO, i.e., spilanthol, d-limonene, and scopoletin, followed Lipinski's rule or “rule of five,” as the log *P*, molecular weight (MW), hydrogen bond acceptors (HBA), and hydrogen bond donors (HBD) were 3.39; 2.72; 1.86 (log *P* ≤ 5), 221.34; 136.23; 192.17 (MW ≤ 500), 1; 0; 4 (HBA ≤ 10) and 1.0; 1 (HBD ≤ 5), HBA ≤ 10) and 1.0; 1 (HBD ≤ 5), respectively, confirming their bioavailability.

MW is an important aspect of the drug's therapeutic activity; if it increases beyond a certain limit, the volume of compounds also increases correspondingly, which in turn affects the drug's activity [20, 21]. Drug with MW < 500 are easily transported, diffused, and absorbed compared to heavy molecules. Although some drugs with a MW higher than that established by Lipinski's rule of five (<500 Da) violate this principle and may have a good lipid solubility profile. The analyzed compounds spilanthol, d-limonene, and scopoletin presented a MW of 221.34, 136.23, and 192.17, respectively. This aspect reinforces the pharmacological potential and low toxicity of the extract and its phytochemicals.

### 2.3. Weight and Metabolic Alterations

**Figure 3:**
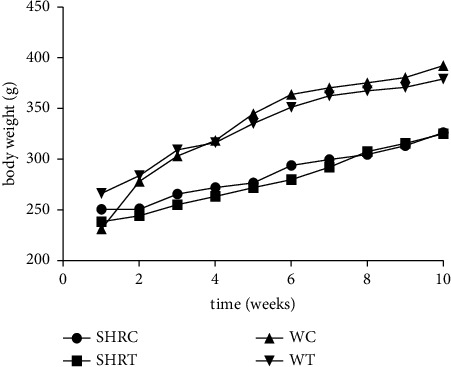
Weight assessment of the animals submitted to treatment via gavage with the hydroethanolic extract of flowers of *A. oleracea*; WC = control Wistar, WT = treated Wistar, SHRC = control spontaneously hypertensive rats, SHRT = treated spontaneously hypertensive rats where controls were treated with 100 *μ*L of saline, and those treated with 100 mg/kg of EHFAo. Data are expressed as the mean ± standard deviation (SD) of the control and treated groups. Statistical analysis was performed using one-way ANOVA followed by Sidak's multiple comparison test ^*∗∗*^*p* < 0.01 when compared to controls.

No relevant differences in the body mass progression of the animals treated with EHFAO (WRT and SHRT, WR and SHR, respectively) were found compared to the respective controls (WRC and SHRC, WR and SHR, respectively) (Figure 3). After 60 days of treatment, the weight gains measured in the last week of treatment: 396 ± 42 g (WC), 374 ± 20 g (WT), 326 ± 19 g (SHRC), and 314 ± 15 g (SHRT), were linearly proportional to the aging process, indicating the absence of toxicity. Furthermore, no deaths were observed during the treatment period.

**Figure 4:**
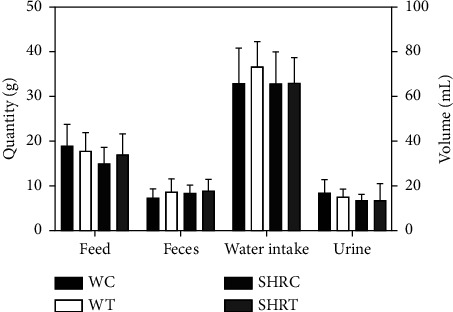
Analysis of physiological parameters after metabolic cage. WC = control Wistar, WT = treated Wistar, SHRC = spontaneously hypertensive control rats, SHRT = spontaneously hypertensive rats treated where controls were treated with 100 *μ*L saline, and those treated with 100 mg/kg of the hydroethanolic extract of flowers of *A. oleracea*. Data are expressed as the control and treated groups' mean ± standard deviation (SD). Statistical analysis was performed using two-tailed ANOVA followed by Sidak's multiple comparison test ^*∗*^*p* < 0.05 compared to controls.

Behavioral observation helps identify changes in the physiological patterns. Some signs such as body reduction, weight gain, and changes in the food intake are important observations, as they may indicate a sign of toxicity to the evaluated substances [22, 23]. After a 12-hour stay in the metabolic cage, no significant variations in these parameters were found in the different groups. Although a slight increase in water consumption among the WR was noticed, the difference was not statistically significant colocar (*p*>0.05) (Figure 4).

Rocha et al. [14] evaluated the reproductive toxicity of the hydroethanolic extract of *A. oleracea* flowers in female WR in 2 doses (88.91 mg/kg and 444.57 mg/kg), the first slightly lower and the second 4 times higher than that used in our study. They reported an increment in water consumption during the two weeks of observation, which could be attributed to the increased sialorrhea triggered by spilanthol [24]. The lack of difference in food consumption, urine volume, and amounts of feces observed among the groups corroborate the findings of Rocha et al. [14].

**Figure 5:**
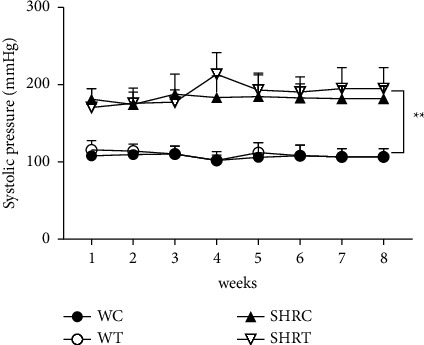
Systolic blood pressure values of rats of all strains in the group were measured weekly by tail plethysmography. Data were expressed as mean ± standard error of the mean. WC = Wistar control, WT = treated Wistar, SHRC = spontaneously hypertensive rat control, and SHRT = treated spontaneously hypertensive rat. WC and SHRC = 100 *μ*L saline; WT and SHRT = 100 mg/kg EHFAO. Statistical analysis of variance; two-way ANOVA followed by multiple comparisons using GraphPad Prism 6.0. ^*∗∗*^*p* < 0.01, when the group was compared with the WC group.

Even though it has been reported previously in the literature that spilanthol presented vasodilation activity *in vitro* [20], the oral administration of EHFAO did not promote any significant change in the blood pressure levels of the WR nor SHR (Figure 5). After physiological analysis, the effect on hepatic and renal biochemical markers was evaluated.

### 2.4. Serum and Urine Biochemical Parameters

**Figure 6:**
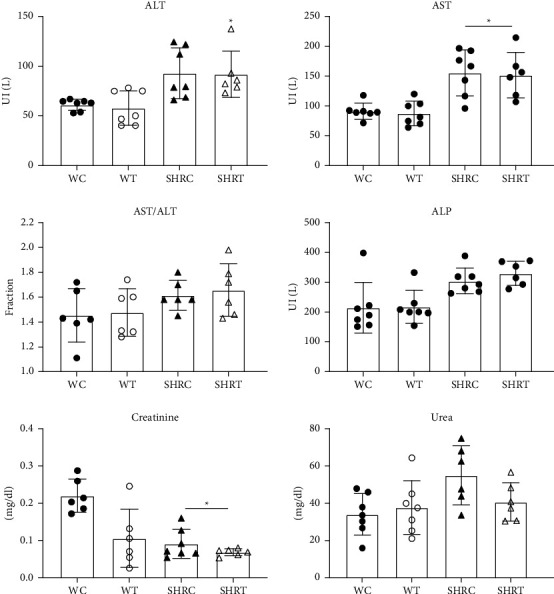
Hepatic and renal biochemical markers after oral administration of hydroethanolic extract of flowers of *A. oleracea* in WC = Wistar control (WC), treated Wistar (WT) rats, spontaneously hypertensive control (SHRC) rats, and spontaneously hypertensive treated (SHRT) rats. Data are expressed as the mean ± standard error (SD); statistical analysis of variance; one-way ANOVA followed by multiple comparisons using GraphPad Prism 6.0. ^*∗*^*p* < 0.05, when the group was compared with the WC group.

The oral treatment of male WR with EHFAO did not interfere in the serum levels of aspartate aminotransferase (AST), alanine aminotransferase (ALT), alkaline phosphatase (ALP), and urea, when compared to the control group, while creatinine was reduced when the group SHR was compared with the WR control group (*p* < 0.05) (Figure 6).

Regarding the male SHR, the treatment did not alter any of the biochemical markers analyzed. The results in female WR by Rocha et al. [14] showed that 10% or 50% of the LD_50_ (88.91 and 444.57 mg/kg, respectively) decreased AST, without statistical significance. In the same study, no changes in the serum ALT, creatinine, or urea were identified, while the AST levels significantly decreased in the lower dose administered (88.91 mg/kg).

The discrepancy in the results for the serum values in the WR treated with EHFAO compared to the study by Rocha et al. [14] could be explained by differences in the extract composition, sex of the animals, and mainly, by the treatment extension. In the study by Rocha et al. [14], the estimated concentration of spilanthol was approximately 81%, while in this study, it reached over 97% in the extract used. Rocha et al. [14] used female animals, while male animals were used in this study. Most importantly, while in that study, the animals were treated for 21 days, remover and in this study, the treatment was extended for 60 days. Lineage differences could further explain the differences found between WR and SRH.

**Figure 7:**
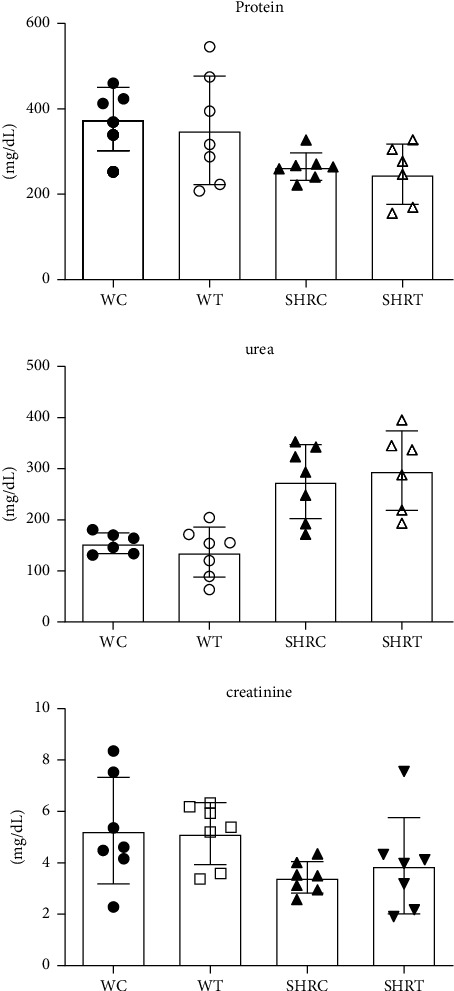
Biochemical analysis of urine after treatment with the hydroethanolic extract of flowers of *A. oleracea* was collected at the end of the 12 hours in a metabolic cage. Data are expressed as the mean ± standard error (SD). Statistical analysis of variance; one-way ANOVA followed by multiple comparisons using GraphPad Prism 6.0. ^*∗*^*p* < 0.05, when the group was compared with the WC group.

No protein, urea, or creatinine differences were found in the urine levels (Figure 7). Despite the observed changes, the values remain within the reference range [25].

### 2.5. Histopathological Analysis

**Figure 8:**
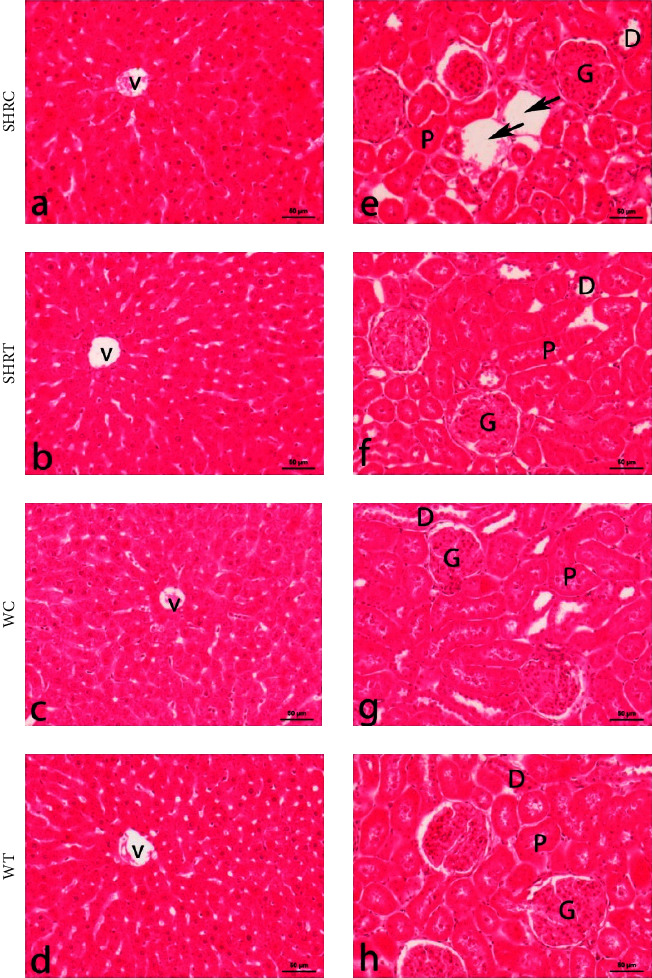
Histopathological analysis of the liver and kidney of the SHR and WT groups. Livers (a–d) and kidneys (e–h). Morphological aspects of the liver of rats treated with the hydroethanolic extract of flowers of *A. oleracea* in both strains (b, d) were similar to their respective control groups (a, c). Likewise, no morphological changes were obtained in the kidneys of SHRT and WT rodents (f, h). V = centrilobular vein of the liver; G = renal glomerulus and D = distal convoluted tubule; P = proximal convoluted tubule; swollen lymphatic vessels present only in the control SHR (arrows).

The effect of EHFAO treatment was also evaluated on the animals' liver and kidney histopathological patterns. No changes were observed in the analysis of both lineages treated with EHFAO (Figure 8). This aspect corroborates with a previous study which evaluated the toxicity of the ethanolic extract of *A. oleracea* in mice (5, 50, and 500 mg/kg), which also did not observe histopathological alterations in the target organs [26]. Chakraborty et al. [27] and Sharma et al. [28] administered oral doses of 2,000 and 3,000 mg/kg of the extract of *A. oleracea* and did not observe behavioral alterations, toxic effects, or mortality, reinforcing the absence of toxicity found.

Divergent results were found in the studies of De Souza et al. [6] using the Zebrafish model. The authors used the hydroethanolic extract of *A. oleracea* flowers to assess the acute toxicity in these animals in two different ways: by immersion and after oral administration. Behavioral changes and death, with a mean oral lethal dose calculated at 148.42 mg/kg, were observed, while the immersion in the treatment caused histopathological changes in the liver, intestine, and kidneys.

The same author [5] evaluated the effect of the hydroethanolic extract of *A. oleracea* flowers on the Zebrafish fertility. The treatment did not cause harmful changes in the gonadal tissues of the progenitors or fertility alterations in the adult animals but caused some potentially teratogenic effects in embryos, which could be related to spilanthol metabolites, according to the *in silico* evaluation. Ponpornpisit et al. [29] also evaluated the toxicity of *A. oleracea* extract in embryos; however, using the aqueous extract from leaves and without providing the chemical composition make comparisons limited. When administered up to 20%, the extract was not lethal, but it was sublethal at 10% and could induce malformations at 20%.

The toxicity observed in some zebrafish parameters may be due to interspecific and ontological differences to adult rodents, as the extracts of *A. oleracea* have demonstrated to be safe even at high doses tested in the aforementioned studies.

### 2.6. *In Silico* Toxicological and Pharmacokinetic Evaluation

In view of the limitations to compare similar experimental models *in vivo*, other tools such as *in silico* modeling can be used to predict and validate these findings.

The major compounds found in the EHFAO showed potential drug-likeness, according to the Lipinski parameters (Table 2). This rule, also called the rule of 5, is widely used to determine the pharmacokinetic properties of compounds and also to assess the permeability and solubility of drugs administered orally [30].

The toxicity assessment was evaluated using the Protox online server to obtain the values of LD_50_, risk class, and molecular target toxicity probabilities. The toxicity and probability predictions, used to identify the toxic target of compounds present in the EHFAO, are also shown in Table 3. The result of the predictions represents the probability of each molecule to cause toxicity over specific targets.

The compounds tested were found to have a small probability of being toxic to the selected targets, marked as inactive in the predictions. Only scopoletin was active for carcinogenicity (53%), immunogenicity (54%), and aryl hydrocarbon receptor (AhR) (51%) still, with reasonable probability. Nonetheless, this molecule was found as traces in the EHFAO composition.

As for LD_50_, the class V risk may be harmful if ingested (2,000 < LD_50_ ≤ 5,000). The results found for the compounds, spilanthol (LD_50_ = 4,387 mg/kg), d-limonene (LD_50_ = 4,400 mg/kg), and scopoletin (LD_50_ = 3,800 mg/kg) (Table 3), indicate that the dose administered in this study would not be toxic to the animals.

The results of the toxicological predictions (mutagenicity and carcinogenicity) of the EHFAO metabolites are described in Table 4. Predictions of mutagenicity were evaluated using the Ames test, while carcinogenicity was evaluated for rats and mice. All metabolites showed mutagenic predictions. As for carcinogenicity, all metabolites were positive for rats, the animal model used in this study.

Human intestinal absorption (HIA) and *in vitro* cell penetrability using the Caco-2 cell model was used to describe the intestinal absorption. The three metabolites were classified as well absorbable (HIA ≥ 70%): spilanthol (97.23%), d-limonene (100%), and scopoletin (93.92%) (Table 4). Regarding the cellular penetrability in Caco-2, two metabolites showed medium permeability (4–70 nm/s): spilanthol (49.321) and d-limonene (23.6317), whereas scopoletin (0.27754) showed low permeability (<4 nm/s) (Table 4).

Exposure of the gastrointestinal tract to toxic substances or xenobiotic compounds can damage its mucosa and impair its physiology and function [31]. For instance, the bioavailability of drugs orally administered is directly linked to significant gastrointestinal absorption, as the movement of a molecule through the organism is affected by its diffusion [28]. In this study, all compounds meet the rules, suggesting that these compounds are suitable as potential drugs. This is in line with Veryser et al. [32], who showed that spilanthol permeated Caco-2 cells *in vitro* from the apical to the basolateral side and vice versa, which was further confirmed *in vivo* in the intestinal lumen of rats.

Regarding the distribution properties, the following parameters were evaluated: binding to plasma proteins (BPP (%)) and interaction with P-glycoprotein (P-GP). Plasma drug-protein binding may affect the drug half-life. Also, the bound moiety can act as a chemical reservoir for the drug, as the bound drug will be released to maintain the equilibrium, while the unbound moiety will be metabolized and excreted from the body [33]. The calculated BPP values obtained were spilanthol (99.528%), d-limonene (100%), and scopoletin (29.418%). Therefore, EHFAO and the metabolites, spilanthol and d-limonene, were classified as highly bound, while scopoletin was considered to be weakly bound to plasma proteins (PPB < 90%) (Table 4). Neither *in vitro* nor *in vivo* studies were found in the literature exploring this property to the date.

The *in-silico* study showed that the metabolites spilanthol and d-limonene were likely to be P-GP inhibitors, while scopoletin was not. Regarding the metabolism, EHFAO metabolites presented a potential inhibition over the CYP450 system (Table 4). The lipophilic nature of this molecule favors transport. Boonen et al. [22] showed that the ethanolic extract of *A. oleracea* was able to permeate the oral mucosa *ex-vivo*.

The blood-brain barrier (BBB) penetration rate (Table 4) is another crucial aspect. For spilanthol (6.68585) and d-limonene (8.27823), the BBB was found >1, indicating that these molecules can permeate through the BBB, in agreement with Veryser et al. [33], who indicated that spilanthol can quickly cross the BBB in mice due to its lipophilic nature.

The human ether-a-go-go (hERG) related gene is encoded for a protein that forms a voltage-gated potassium ion channel in the heart and nervous system. This channel is essential for repolarization during the cardiac action potential. Conductance changes of this channel, especially blockage, can lead to an impaired action potential [34]. Due to the importance in regulating cardiac action potential, drugs that can interact with hERG are currently being withdrawn from the market, as this can result in arrhythmia and sudden death [34, 35]. In this study, spilanthol and d-limonene were found to have a medium risk of interacting with hERG, while scopoletin presented a low risk.

Regarding the renal clearance, EHFAO major compounds showed the following results in the PMDCK system (nn/sec): D-limonene (238.434) showed high permeability (>70 nn/sec), while spilanthol (5.57533) and scopoletin (67.4595) showed medium permeability (4–70 nn/sec) (Table 4). Therefore, EHFAO and its phytochemicals showed high and medium permeability in PMDCK cells from the rat kidney, so it is reasonable to assume that these compounds would not affect glomerular filtration and renal clearance [36].

#### 2.6.1. Prediction of Lipophilicity and Solubility

The solubility in pure water is a key property for drug development as it is directly related to the compound's pharmacokinetics. According to Di et al. [37], the 1-octanol/water partition coefficient log *P* is used as a parameter to express the lipophilicity of a given compound [38]. The predisposition to decompose a compound in a nonpolar or aqueous environment can be directly affected by its lipophilicity. Therefore, the greater the lipophilicity of a compound, the greater its permeability, protein binding, volume of distribution, and renal excretion [37].

The EHFAO compounds, spilanthol, d-limonene, and scopoletin, had logP values equal to 3.55, 2.72, and 1.86, respectively (Table 5). In fact, only positive logP values in the range of 0.97–4.57 were found in this study, indicating reasonable lipophilicities. According to Sepay et al. [39], water solubility is also an important requirement for the drug candidate administration, both orally or parenterally, as a sufficient amount of active pharmaceutical ingredients must be administered in small volumes.

**Figure 9:**
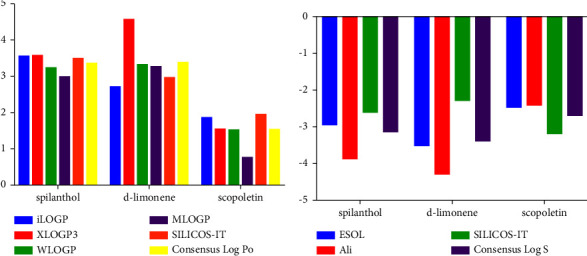
Log *P* (a) and Log *S* (b) values were predicted using different methodologies for the pivotal molecule and the hydroethanolic extract of flowers of *A. oleracea* compounds.

Spilanthol, d-limonene, and scopoletin presented logS values of −2.93, −3.50, and −2.46, respectively (Figure 9). According to Sepay et al. [39], log *S* values between −4 and −6 indicate a moderate solubility, −2 to −4 indicate good solubility, and greater than −6 indicate low solubility. Therefore, EHFAO compounds are likely to be water-soluble and promising for oral administration.

#### 2.6.2. Prediction of Biological Activities *In Silico*

The PASS server was used to obtain the predicted biological activity profile for the three major compounds found in the EHFAO. The biological potential of a molecule is evaluated in this software, similar to a drug complying with the Pa and Pi criteria. The chance of a compound being active (Pa) and being inactive (Pi) for such activities is estimated. The selection of results was based on Pa values >0.7 and Pi values <0.05, with the probability of being active or inactive, respectively. Some compounds were found active for the same biological activity (Table 6).

Spilanthol presented the highest value and the most promising results among the EHFAO compounds for phobic disorder treatment (0.821Pa–0.026Pi) and as a mucomembranous protector (0.801Pa–0.018Pi). For d-limonene, the most relevant biological activities were antieczematic (0.896Pa–0.005Pi) and testosterone 17beta-dehydrogenase (NADP+) inhibition (0.753Pa–0.038Pi). Finally, for scopoletin, the most relevant biological activities found were ubiquinol-cytochrome-c reductase inhibition (0.829Pa–0.023Pi) and as a TP53 expression enhancer (0.805Pa–Pi 0.010Pi).

TP53 gene mutations are the most common genetic alterations in human malignancies. The three compounds showed a high probability of being active over the TP53 expression and ubiquinol-cytochrome-c reductase inhibition activities. Overexpression of the p53 protein has been reported frequently in all types of skin cancer and bladder [40–42]. The treatment with the extract of *Heliopsis longipes* SF Blake (Asteraceae), in which spilanthol is also the major phytochemical, inhibited breast cancer angiogenesis and could increase p53 levels, the latter related to apoptosis, resulting in a reduction in the tumor's size [43]. Another study investigated the effect of D-limonene on BGC-823 gastric cancer cells, using p53 expression, showing also an apoptotic cytotoxic effect [44]. The modulation of p53 by scopoletin is related to the induction of autophagy, showing the affinity of these compounds with TP53 expression enhancers [45].

The identification of these bioactive molecules, stored in a database and discovered through computational techniques (*in silico*), helps in the development of novel drugs, providing information as candidates for new treatments. Accordingly, the *in-silico* evaluation carried out in this study confirmed the oral bioavailability of these compounds in the administration of EHFAO and predicted the possible hazardous effects and open new studies' perspectives, to test the isolated compounds directly with the predicted biological activities.

## 3. Materials and Methods

### 3.1. Phytochemistry

#### 3.1.1. Chemicals

Ethyl alcohol containing at least 92.55°GL, acetonitrile HPLC gradient (≥99.9%) grade, and methanol were obtained from Labbox Labware S.L (Spain), while acetonitrile HPLC supra gradient grade and formic acid were obtained from Scharlab S.L (Spain). All the other reagents were of pure grade and used as received.

#### 3.1.2. Plant Material

Fresh flowers of *Acmella oleracea* (L) R. K. Jansen were obtained in the agricultural center of Fazendinha District (Lat. 00°02′30.40″S/Long. 51°06′37.5″W), in the city of Macapá, Brazil, between April and May 2018. The plant exsiccate was deposited at the Herbarium Amapaense (HAMAB/*Instituto de Pesquisas Científicas e Tecnológicas do Estado do Amapá-IEPA*) under the registration number: Brazil. Map for: 05. VII.2019, P. Peretti, 001/002, HAMAB.

#### 3.1.3. Extraction Procedure

The hydroethanolic extract of *A. oleracea* flowers (EHFAO) was obtained by static maceration. The flowers were dried in a laboratory oven (Quimis Q31, Quimis, Diadema, São Paulo, Brazil) at –50°C for 3 days, grounded in a knife mill (Quimis Q298 A, Quimis, Diadema, São Paulo, Brazil), weighed, and transferred at 4% w/v into a dark glass bottle containing ethanol 92° for 3 days. This process was repeated until reaching the extractive exhaustion, marked by the solvent clearness. The macerate was gravity filtered (Qualy-Prolab, Pro Lab Inc., Richmond Hill, Ontario, Canada) and rotaevaporated at 50°C to remove the solvent. The residue obtained was placed in clean amber glass flasks previously weighed. The extractive yield (%) was calculated by comparing the mass of the crude extract with the mass of the dry residue before the extractive procedures.

#### 3.1.4. Ultraperformance Liquid Chromatography Analysis

The metabolites quantification followed the method used by Peretti et al. [21]. The dry extract of *A. oleracea* was carefully weighed (–2 mg), dissolved in 95% ethanol at a ratio of 1 : 140, filtered through a syringe filter (GHP 0.2 mm Acrodisc®), and subjected to UHPLC-ESI-QToF-MS/MS. A UHPLC system (Elute, Bruker Daltonics, Billerica, MA, USA) coupled to an ESI-Q-ToF mass de detector (timsToF, Bruker Daltonics, Billerica, MA, USA) analysis. A reversed-phase C18 column (100 × 2.1 mm, with a particle size of 2.6 mm, 100 Å pore length, and a 0.5 mm × 0.004 porosity in-line filter), was used (Kinetex, Phenomenex, Torrance, CA, USA). The mobile phase gradient ranged from 95% A and 5% B to inverse concentrations, where A was the water containing 0.1% (v/v) of formic acid and B was the acetonitrile containing 0.1% (v/v) of formic acid. Mass spectrometric detection was in the positive mode with a scanning range of 50–1000 m/z, while the flow rate used was 0.3 ml/min, with the injection volume of 2 ml and the total analysis time of 16 min. The capillary temperature was 200°C, the gas sheath pressure was 2.5 Bar, and the capillary and tubular lens voltage was 4500 V.

### 3.2. Animal Experimentation

#### 3.2.1. Ethical Statement and Experimental Protocol

The project was approved by the Ethics Committee on the Use of Animals of the Federal University of Goiás (CEUA-UFG 065/19). The animals were donated by the Central Animal Facility of the same university (UFG-Campus Samambaia).

Twenty male Wistar (WR) (*Rattus novergicus*) and twenty male spontaneously hypertensive (SHR) rats, both 9 weeks old, were used in this study. The animals were allocated according to the following groups: WRC and WRT (WR control and test, respectively); SHRC and SHRT (SHR control and test, respectively). The mean initial weights of each group were WRC (335.9 g), WRT (334.2 g), SHRC (285.3 g), and SHRT (279.5 g). The animals were divided into four groups by simple randomization (two controls and two treated with EHFAO-10 animals/strain) and kept in plastic cages (5 animals per cage). They were maintained at a temperature of 25°C with a 12 h dark/light cycle (lights on at 7 a.m.). The animals in the EHFAO group received 100 mg/kg/daily of EHFAO extract diluted in saline solution by oral gavage for 60 days, according to the protocol adapted from Rocha et al. [5]. The dose was adjusted weekly according to the body weight. The control group received only saline solution during the same period.

#### 3.2.2. Metabolic Cage

In the final phase of the treatment, the rats were housed for 12 h in metabolic cages with a controlled temperature of 25°C, with a dark cycle. Water and food were provided *ad libitum*. The volume of water intake, volume of urine excretion, and food intake were measured. Urine samples were collected to measure proteins, urea, and creatinine.

On the last day, the rats were weighed and anesthetized with a ketamine/xylazine solution (100 mg/kg and 10 mg/kg) intraperitoneally and blood samples from the inferior vena cava were collected for biochemical analysis, followed by euthanasia and collection of the kidneys and liver.

#### 3.2.3. Biochemical Analysis

The glomerular filtration rate (GFR) was determined colorimetrically by creatinine clearance (CCr) in mL/min. Blood urea and creatinine concentrations were also measured by colorimetric methods using a commercial kit (Bioclin®) (BELphotonics 1105 spectrophotometer, *λ* = 600 nm). Serum aspartate aminotransferase (AST) and alanine aminotransferase (ALT) levels, together with the calculation of the ALT/AST ratio and alkaline phosphatase (ALP), were used to assess the liver function.

#### 3.2.4. Histopathological Preparation

The organs of 5 animals/group were fixed by immersion in metacarn (60% methanol, 30% chloroform, and 10% acetic acid) for 4 h at 4°C. After that, they were dehydrated in an increasing series of 60, 70, 80, 90, and 100% ethanol. Subsequently, they were clarified in xylene, embedded in paraplast (Histosec, Merck), and sectioned at 5 *μ*m using a Leica microtome (Leica RM2155). The selected sections were stained with hematoxylin-eosin (HE), and the samples were analyzed using a Zeiss Axioscope A1 light microscope.

#### 3.2.5. Statistical Analysis

The obtained data were submitted in pairs (test and control of the same lineage) to analysis of variance (one-way ANOVA) with Tukey's post-hoc test when appropriate. A significance level of 5% (*p* < 0.05) was considered significant. Analyses comparing only treated and control groups of the same strain were performed using Student's *t*-test; significance was indicated when *p* < 0.05.

### 3.3. *In silico* Predictive Analysis

#### 3.3.1. Toxicological and Pharmacokinetic Parameters of the Major Compounds

The ProTox [46, 47] platform was used to obtain values for LD_50_, risk class, and toxic probability of the molecular targets. The PreADMET (https://preadmet.qsarhub.com/toxicity/) was used to predict the toxicological and pharmacokinetic properties: mutagenicity, carcinogenicity, *in vitro* human ether-a-go-go related gene channel inhibition (hERG), human intestinal absorption (HIA), cell penetrability *in vitro* using the Caco-2 cell model, plasma protein binding (PPB (%), brain-blood partition coefficient BBB (Cbrain/Cblood), interaction with P-glycoprotein (P-GP), CYP450, and Madin–Darby canine kidney cells PMDCK (nm/sec).

#### 3.3.2. Prediction of Lipophilicity and Solubility

The prediction of lipophilicity and water solubility was evaluated by SwissADME software [47, 48] and expressed through log *P* and log *S* values, based on free solvation energies in 1-octanol and water calculated by the generalized Born model and access to the solvent of surface area (GB/SA). It has a performance equal to or greater than six well-established predictors.

#### 3.3.3. Biological Activities

The probability of biological activities was assessed using the PASS software package [49, 50]. This platform is capable of predicting up to 2,000 biological activities for chemical compounds with an accuracy of 70–80%. The result is expressed as the probability of being active (Pa) and being inactive (Pi) for each investigated target.

#### 3.3.4. Oral Bioavailability (Rule of Five: Lipinski)

**Figure 10:**
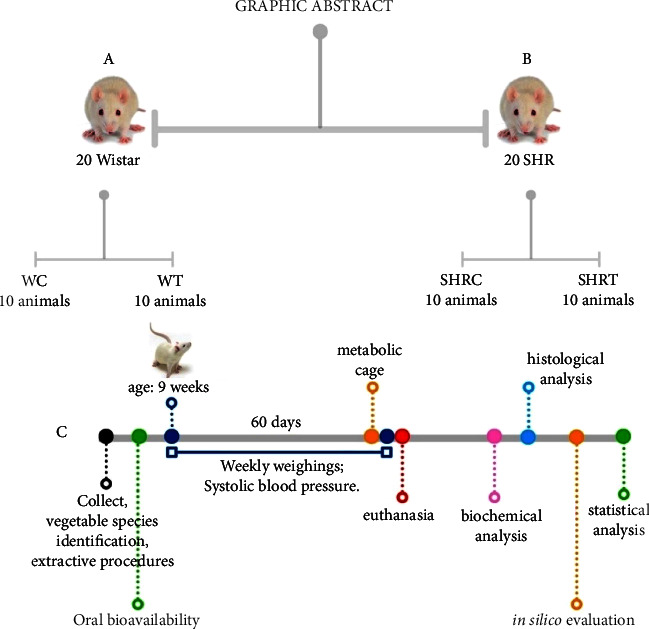
Experimental design. (A) 20 Wistar rats (10 control group; 10 hydroethanolic extract of flowers of the *A. oleracea*-treated group). (B) 20 SHR (10 control group, 10 hydroethanolic extract of A. *oleracea* flowers-treated group group. (C) Hydroethanolic extract of A. *oleracea* flowers administration via gavage. Timeline with the order analysis: collection, identification, and preparation of the *A. oleracea* extract, oral bioavailability, weekly weighing, pressure measurement, metabolic cage, euthanasia, biochemical analysis, histological analysis, *in silico* evaluation, and statistical analysis.

The compounds identified were searched in the PubChem database [51] to obtain the SMILES code, which was then inserted into the ProTox [52] to calculate the molecular weight (MW), hydrogen bonding acceptors (HBA), hydrogen bonding donors (HBD), and the number of properties of rotational bonds. If a molecule follows Lipinski's rule, it is likely to have good bioavailability and also to be a good therapeutic candidate: i.e., −log *P* shall not be more than 5, the MW below 500 Da, the number of hydrogen bond acceptors ≤10, and the number of hydrogen bond donors ≤5. All methodological steps (experimental and *in silico*) are summarized in Figure 10.

## 4. Conclusions

The ethanolic extract of *A. oleracea* flowers did not cause weight alterations, neither in the blood pressure, plasmatic or urinary levels of AST, ALT, creatinine and creatinine levels in any of the treated animal strains when compared to their respective controls. Moreover, no histopathological alterations were observed in the animals' kidneys and liver, suggesting its safety in the protocol used in this study.

The EHFAO and its major compounds, spilanthol, d-limonene, and scopoletin, presented a good performance *in silico*, showing excellent oral bioavailability, absorption, solubility, and lipophilicity.

This study reinforces the safety on the long-term consumption of *A. oleracea*. In addition, the compounds identified can be selected for future *in vitro* and/or *in vivo* tests, as the results of the prediction of biological activities point a great pharmacological potential for different targets.

## Figures and Tables

**Table 1:** Qualitative and quantitative analysis of the EHFAO. Data were obtained by HPLC-ESI-QTOF-MS/MS identifying spilanthol (N-isobutyl amide-2E, 6Z, and 8E-decatrienoic acid) as the major constituent at a retention time of 6.97 minutes.

Overall yield (%)	Compound	Retention (min)	Standard *m*/*z*	m Sigma	Content (%)	Concentration over ppm ± SD
1.82	Spilanthol	6.97	222.1847	0.75	97.7	50 ± 15

*m*/*z*: charge/mass ratio; min: minutes; SD: standard deviation. Source: author.

**Table 2:** Oral bioavailability properties and application of the Lipinski rule for the phytochemicals found in the hydroethanolic extract of flowers of *A. oleracea*.

Compounds	LogP^2^	MW^1^	HBA^3^	HBD^4^	Smiles code^5^
Spilanthol	3.39	221.34	1	1	CC=CC=CCCC=CC(=O)NCC(C)C
D-limonen	2.72	136.23	0	0	CC1=CCC(CC1)C(=C)C
Scopoletin	1.86	192.17	4	1	COC1=C(C=C2C(=C1)C=CC(=O)O2)O

^1^Molecular weight, ^2^logarithm of the partition between of n-octanol and water phases, ^3^hydrogen bond acceptor, ^4^hydrogen bond donor, and ^5^Smiles code obtained in PubChem.

**Table 3:** Toxicity and probability predictions for the toxic targets of spilanthol, D-limonene, and scopoletin found in the hydroethanolic extract of flowers of *A. oleracea* and toxicity model report.

Compounds	Spilanthol		D-limonene		Scopoletin	
LD_50_ toxic (mg/kg)	4,378		4,400		3,800	
Risk class	V		V		V	

Toxicity model report	Prediction	Probability (%)	Prediction	Probability (%)	Prediction	Probability (%)

Hepatotoxicity	Inactive	86	Inactive	76	Inactive	69
Carcinogenicity	Inactive	61	Inactive	65	Active	53
Immunotoxicity	Inactive	98	Inactive	95	Active	54
Mutagenicity	Inactive	80	Inactive	97	Inactive	56
Cytotoxicity	Inactive	75	Inactive	82	Inactive	91
Aryl hydrocarbon receptor (AhR)	Inactive	97	Inactive	1.0	Active	51
Androgen receptor (AR)	Inactive	99	Inactive	99	Inactive	99
Androgen receptor ligand binding domain (AR-LBD)	Inactive	99	Inactive	100	Inactive	78
Aromatase	Inactive	98	Inactive	99	Inactive	89
Estrogen receptor alpha (ER)	Inactive	93	Inactive	84	Inactive	71
Estrogen receptor ligand binding domain (ER-LBD)	Inactive	99	Inactive	100	Inactive	70
Peroxisome proliferator activated receptor gamma (PPAR-gamma)	Inactive	99	Inactive	100	Inactive	95
Nuclear factor (erythroid-derived 2)-like 2/antioxidant responsive element (nrf2/ARE)	Inactive	98	Inactive	98	Inactive	96
Heat shock factor response element (HSE)	Inactive	98	Inactive	98	Inactive	96
Mitochondrial membrane potential (MMP)	Inactive	97	Inactive	100	Inactive	53
Phosphoprotein (tumor supressor) p53	Inactive	99	Inactive	100	Inactive	87
ATPase family AAA domain-containing protein 5 (ATAD5)	Inactive	99	Inactive	100	Inactive	75

Class I: fatal if swallowed (LD_50_ ≤ 5); Class II: fatal if swallowed (5 < LD_50_ ≤ 50); Class III: toxic if swallowed (50 < LD_50_ ≤ 300); Class IV: harmful if swallowed (300 < LD_50_ ≤ 2000); Class V: may be harmful if swallowed (2000 < LD_50_ ≤ 5000); Class VI: nontoxic (LD_50_ > 5000).

**Table 4:** Prediction of pharmacokinetics parameters of spilanthol, D-limonene, and scopoletin found in the hydroethanolic extract of flowers of *A. oleracea*.

Compounds	Mutagenicity	Carcinogenicity	hERG	HIA (%)	Caco-2(nm/s)	Distribution PPB (%)	P-GP	BBB Cbrain/Cblood (Cbrain/Cblood)	CYP450	Excretion PMDCK (nm/s)
Spilanthol	Mutagen	Mouse positive	Medium risk	9.723.631	49.321	9.952.837	Inhibitor	668.585	Non	557.533
		Rat positive								

D-limonene	Mutagen	Mouse negative	Medium risk	100	23.631	100	Inhibitor	827.823	Non	238.434
		Rat positive								

Scopoletin	Mutagen	Mouse negative	Low risk	9.392.356	0.27754	2.941.838	Non	0.644081	Non	674.595
		Rat positive								

Values obtained by PREADMET. Human ether-a-go-go related gene channel inhibition (hERG), human intestinal absorption (HIA), cell penetrability *in vitro* using the Caco-2 cell model, plasma protein binding (PPB (%), interaction with P-glycoprotein (P-GP), brain-blood partition coefficient BBB (Cbrain/Cblood), CYP450, and Madin-Darby canine kidney cells PMDCK (nm/sec).

**Table 5:** Prediction of pharmacokinetics parameters of the phytochemicals found in the hydroethanolic extract of flowers of *A. oleracea*.

Lipophilicity	Spilanthol	D-limonene	Scopoletin
Log Po/w (iLOGP)	3.55	2.72	1.86
Log Po/w (XLOGP3)	3.57	4.57	1.53
Log Po/w (WLOGP)	3.23	3.31	1.51
Log Po/w (MLOGP)	2.99	3.27	0.76
Log Po/w (SILICOS-IT)	3.49	2.97	1.94
Consensus log Po/w	3.36	3.37	1.52
*Water solubility*			
Log *S* (ESOL)	−2.93	−3.50	−2.46
Log *S* (Ali)	−3.87	−4.29	−2.39
Log *S* (SILICOS-IT)	−2.58	−2.26	−3.17
Consensus log *S*	−3.12	−3.35	−2.67

**Table 6:** Prediction of biological activity of promising the major compounds found in the hydroethanolic extract of flowers of *A. oleracea* via PASS online.

Prediction of biological activity	Spilanthol		D-limonene		Scopoletin	
	Pa	Pi	Pa	Pi	Pa	Pi
Respiratory analeptic	—	—	0.716	0.014	—	—
TP53 expression enhancer	—	—	—	—	0.805	0.010
Antieczematic	0.703	0.044	0.896	0.005	—	—
CYP2J2 substrate	0.720	0.028	—	—	—	—
Fatty-acyl-CoA synthase inhibitor	0.730	0.009	—	—	—	—
Testosterone 17beta-dehydrogenase (NADP+) inhibitor	0.751	0.039	0.753	0.038	0.707	0.053
Ubiquinol-cytochrome-c reductase inhibitor	0.765	0.045	0.707	0.066	0.829	0.023
Chymosin inhibitor	0.796	0.021	—	—	—	—
Saccharopepsin inhibitor	0.796	0.021	—	—	—	—
Acrocylindropepsin inhibitor	0.796	0.021	—	—	—	—
CYP2J substrate	0.796	0.022	0.747	0.035	—	—
Mucomembranous protector	0.801	0.018	—	—	—	—
Phobic disorders treatment	0.821	0.026	—	—	—	—

## Data Availability

The data used to support the findings of this study are available from the corresponding author upon request.

## References

[1] Dallazen J. L., Maria-Ferreira D., da Luz B. B. (2020). Pharmacological potential of alkylamides from Acmella oleracea flowers and synthetic isobutylalkyl amide to treat inflammatory pain. *Inflammopharmacology*.

[2] Huang W.-C., Peng H.-L., Hu S., Wu S.-J. (2019). Spilanthol from traditionally used spilanthes acmella enhances AMPK and ameliorates obesity in mice fed high-fat diet. *Nutrients*.

[3] Freitas Blanco V. S. D., Michalak B., Zelioli I. A. M. (2018). Isolation of spilanthol from Acmella oleracea based on Green Chemistry and evaluation of its in vitro anti-inflammatory activity. *The Journal of Supercritical Fluids*.

[4] Balieiro O. C., da Silva Pinheiro M. S., Silva S. Y. S. (2020). Analytical and preparative chromatographic approaches for extraction of spilanthol from Acmella oleracea flowers. *Microchemical Journal*.

[5] de Souza G. C., Viana M. D., Goés L. D. M. (2020). Reproductive toxicity of the hydroethanolic extract of the flowers of Acmella oleracea and spilanthol in zebrafish: in vivo and in silico evaluation. *Human & Experimental Toxicology*.

[6] De Souza G. C., Matias Pereira A. C., Viana M. D. (2019). Acmella oleracea (L) R. K. Jansen reproductive toxicity in zebrafish: an in vivo and in silico assessment. *Evidence-based Complementary and Alternative Medicine*.

[7] Chan E. W. C., Wong S. K. (2015). Phytochemistry and pharmacology of ornamental gingers, Hedychium coronarium and Alpinia purpurata: a review. *Journal of Integrative Medicine*.

[8] Rautio J., Kumpulainen H., Heimbach T. (2008). Prodrugs: design and clinical applications. *Nature Reviews Drug Discovery*.

[9] de Sá Hyacienth B. M., Tavares Picanço K. R., Sánchez-Ortiz B. L. (2020). Hydroethanolic extract from Endopleura uchi (Huber) Cuatrecasas and its marker bergenin: toxicological and pharmacokinetic studies in silico and in vivo on zebrafish. *Toxicology Reports*.

[10] Calzada M. S. (1988). *Frutales y Hortalizas Promisorios de La Amazonia*.

[11] Costa O. A., Gomes da Cruz J. P. (1947). Plantas medicinais. *Revista da Flora Medicinal*.

[12] Volpato G. T., Francia-Farje L. A. D., Damasceno D. C., Oliveira R. V., Hiruma-Lima C. A., Kempinas W. G. (2015). Effect of essential oil from Citrus aurantium in maternal reproductive outcome and fetal anomaly frequency in rats. *Anais da Academia Brasileira de Ciencias*.

[13] Do Nascimento L. D., de Moraes A. A. B., da Costa K. S. (2020). Bioactive natural compounds and antioxidant activity of essential oils from spice plants: new findings and potential applications. *Biomolecules*.

[14] Da Rocha C. F., De Medeiros Souza Lima Y., Carvalho H. O. (2018). Action of the hydroethanolic extract of the flowers of Acmella oleracea (L.) R. K. Jansen on the reproductive performance of Wistar females rats: a popular female aphrodisiac from the Amazon. *Journal of Ethnopharmacology*.

[15] Ratnasooriya W. D., Pieris K. P. P., Samaratunga U., Jayakody J. R. A. C. (2004). Diuretic activity of Spilanthes acmella flowers in rats. *Journal of Ethnopharmacology*.

[16] Rego C. M. A., Francisco A. F., Boeno C. N. (2022). Inflammasome NLRP3 activation induced by Convulxin, a C-type lectin-like isolated from Crotalus durissus terrificus snake venom. *Scientific Reports*.

[17] Pina J. R. S., Marinho P. S. B., Gomes Júnior P. C. (2022). Antiproliferative, genotoxic activities and quantification of extracts and cucurbitacin B obtained from Luffa operculata (L.) Cogn. *Arabian Journal of Chemistry*.

[18] Lima A. D. M., Siqueira A. S., Möller M. L. S. (2022). In silico improvement of the cyanobacterial lectin microvirin and mannose interaction. *Journal of Biomolecular Structure and Dynamics*.

[19] Spelman K., Depoix D., McCray M., Mouray E., Grellier P. (2011). The traditional medicine Spilanthes acmella, and the alkylamides spilanthol and undeca-2E-ene-8,10-diynoic acid isobutylamide, demonstrate in vitro and in vivo antimalarial activity. *Phytotherapy Research*.

[20] Dubey S., Maity S., Singh M., Saraf S. A., Saha S. (2013). Phytochemistry, pharmacology and toxicology of spilanthes acmella: a review. *Advances in Pharmacological Sciences*.

[21] Peretti P., Rodrigues E. T., de Souza Junior B. M. (2021). Spilanthol content of Acmella oleracea subtypes and their bactericide and antibiofilm activities against Streptococcus mutans. *South African Journal of Botany*.

[22] Boonen J., Baert B., Burvenich C., Blondeel P., De Saeger S., De Spiegeleer B. (2010). LC-MS profiling of N-alkylamides in Spilanthes acmella extract and the transmucosal behaviour of its main bio-active spilanthol. *Journal of Pharmaceutical and Biomedical Analysis*.

[23] Barbosa A. F., Pereira C. D. S. S., Mendes M. F. (2017). Spilanthol content in the extract obtained by supercritical CO2 at different storage times of acmella oleracea L. *Journal of Food Process Engineering*.

[24] Rondanelli M., Fossari F., Vecchio V. (2020). Acmella oleracea for pain management. *Fitoterapia*.

[25] Kurtz D. M., Travlos G. S. (2017). The clinical chemistry of laboratory animals, third edition. *The Clinical Chemistry of Laboratory Animals*.

[26] Nomura E. C. O., Rodrigues M. R. A., Da Silva C. F. (2013). Antinociceptive effects of ethanolic extract from the flowers of Acmella oleracea (L.) R.K. Jansen in mice. *Journal of Ethnopharmacology*.

[27] Chakraborty A., Devi R. K. B., Rita S., Sharatchandra K., Singh T. I. (2004). Preliminary studies on antiinflammatory and analgesic activities of Spilanthes acmella in experimental animal models. *Indian Journal of Pharmacology*.

[28] Sharma V., Boonen J., Chauhan N. S., Thakur M., De Spiegeleer B., Dixit V. (2011). Spilanthes acmella ethanolic flower extract: LC-MS alkylamide profiling and its effects on sexual behavior in male rats. *Phytomedicine*.

[29] Ponpornpisit A., Pirarat N., Suthikrai W., Binwihok A. (2011). Toxicity test of kameng (eclipta prostrate linn.) and kradhuawean (spilanthes acmella (linn.) murr.) to early life stage of zebrafish (Danio rerio). *Thai Journal of Veterinary Medicine*.

[30] Lipinski C. A., Lombardo F., Dominy B. W., Feeney P. J. (1997). Experimental and computational approaches to estimate solubility and permeability in drug discovery and development settings. *Advanced Drug Delivery Reviews*.

[31] Kirchmair J., Göller A. H., Lang D. (2015). Predicting drug metabolism: experiment and/or computation?. *Nature Reviews Drug Discovery*.

[32] Veryser L., Wynendaele E., Taevernier L. (2014). N-alkylamides: from plant to brain. *Functional Foods in Health and Disease*.

[33] Veryser L., Taevernier L., Joshi T. (2016). Mucosal and blood-brain barrier transport kinetics of the plant N-alkylamide spilanthol using in vitro and in vivo models. *BMC Complementary and Alternative Medicine*.

[34] Sliwoski G., Kothiwale S., Meiler J., Lowe E. W. (2014). Computational methods in drug discovery. *Pharmacological Reviews*.

[35] Farias I. V., Faqueti L. G., Noldin V. F. (2018). Cytotoxic phloroglucinol meroterpenoid from Eugenia umbelliflora fruits. *Phytochemistry Letters*.

[36] Collares-Buzato C. B., De Paula Le Sueur L., Da Cruz-Höfling M. A. (2002). Impairment of the cell-to-matrix adhesion and cytotoxicity induced by Bothrops moojeni snake venom in cultured renal tubular epithelia. *Toxicology and Applied Pharmacology*.

[37] Di L., Kerns E. H., Fan K., McConnell O. J., Carter G. T. (2003). High throughput artificial membrane permeability assay for blood-brain barrier. *European Journal of Medicinal Chemistry*.

[38] Daina A., Michielin O., Zoete V. (2014). ILOGP: a simple, robust, and efficient description of n-octanol/water partition coefficient for drug design using the GB/SA approach. *Journal of Chemical Information and Modeling*.

[39] Sepay N. N., Sepay N. N., Al Hoque A., Mondal R., Halder U. C., Muddassir M. (2020). In silico fight against novel coronavirus by finding chromone derivatives as inhibitor of coronavirus main proteases enzyme. *Structural Chemistry*.

[40] Hodgson A., van Rhijn B. W. G., Kim S. S. (2020). Reassessment of p53 immunohistochemistry thresholds in invasive high grade bladder cancer shows a better correlation with TP53 and FGFR3 mutations. *Pathology, Research & Practice*.

[41] Wang X., Sun Q. (2017). TP53 mutations, expression and interaction networks in human cancers. *Oncotarget*.

[42] Weiss J., Heine M., Arden K. C. (1995). Mutation and expression of TP53 in malignant melanomas. *Recent Results in Cancer Research*.

[43] Willig J. B., Salomón J. L. D. O., Vianna D. R. B. (2019). Heliopsis longipes S.F. Blake (Asteraceae) extract causes cell cycle arrest and induces caspase dependent apoptosis against cancer cell lines. *South African Journal of Botany*.

[44] Lu X. G., Feng B. A., Zhan L. B., Yu Z. H. (2003). D-limonene induces apoptosis of gastric cancer cells. *Zhonghua Zhongliu Zazhi*.

[45] Nam H., Kim M. M. (2015). Scopoletin has a potential activity for anti-aging via autophagy in human lung fibroblasts. *Phytomedicine*.

[46] Banerjee P., Eckert A. O., Schrey A. K., Preissner R. (2018). ProTox-II: a webserver for the prediction of toxicity of chemicals. *Nucleic Acids Research*.

[47] Santos K. L. B. D., Cruz J. N., Silva L. B. (2020). Identification of novel chemical entities for adenosine receptor type 2a using molecular modeling approaches. *Molecules*.

[48] Daina A., Michielin O., Zoete V. (2017). SwissADME: a free web tool to evaluate pharmacokinetics, drug-likeness and medicinal chemistry friendliness of small molecules. *Scientific Reports*.

[49] Poroikov V. V., Filimonov D. A., Ihlenfeldt W. D. (2003). PASS biological activity spectrum predictions in the enhanced open NCI Database Browser. *Journal of Chemical Information and Computer Sciences*.

[50] Mascarenhas A. M. S., de Almeida R. B. M., de Araujo Neto M. F. (2020). Pharmacophore-based virtual screening and molecular docking to identify promising dual inhibitors of human acetylcholinesterase and butyrylcholinesterase. *Journal of Biomolecular Structure and Dynamics*.

[51] Kim S., Thiessen P. A., Bolton E. E. (2016). PubChem substance and compound databases. *Nucleic Acids Research*.

[52] Pro-Tox II (2021). ProTox-II - prediction of TOXicity of chemicals. https://tox-new.charite.de/protox_II/index.php?site=compound_search_similarity.

